# Qualitative and Quantitative Evaluation of a Deep Learning-Based Reconstruction for Accelerated Cardiac Cine Imaging

**DOI:** 10.3390/bioengineering12030231

**Published:** 2025-02-24

**Authors:** Junjie Ma, Xucheng Zhu, Suryanarayanan Kaushik, Eman Ali, Liangliang Li, Kavitha Manickam, Ke Li, Martin A. Janich

**Affiliations:** 1 GE HealthCare, Jersey City, NJ 07302, USA; 2 GE HealthCare, Menlo Park, CA 94025, USA; 3 GE HealthCare, Waukesha, WI 53188, USA; 4 GE HealthCare, 80807 Munich, Germany; 5 GE HealthCare, Beijing 100176, China

**Keywords:** accelerated cine imaging, deep learning, retrospective evaluation, cardiac function, free-breathing cine

## Abstract

Two-dimensional (2D) cine imaging is essential in routine clinical cardiac MR (CMR) exams for assessing cardiac structure and function. Traditional cine imaging requires patients to hold their breath for extended periods and maintain consistent heartbeats for optimal image quality, which can be challenging for those with impaired breath-holding capacity or irregular heart rhythms. This study aims to systematically assess the performance of a deep learning-based reconstruction (Sonic DL Cine, GE HealthCare, Waukesha, WI, USA) for accelerated cardiac cine acquisition. Multiple retrospective experiments were designed and conducted to comprehensively evaluate the technique using data from an MR-dedicated extended cardiac torso anatomical phantom (digital phantom) and healthy volunteers on different cardiac planes. Image quality, spatiotemporal sharpness, and biventricular cardiac function were qualitatively and quantitatively compared between Sonic DL Cine-reconstructed images with various accelerations (4-fold to 12-fold) and fully sampled reference images. Both digital phantom and in vivo experiments demonstrate that Sonic DL Cine can accelerate cine acquisitions by up to 12-fold while preserving comparable SNR, contrast, and spatiotemporal sharpness to fully sampled reference images. Measurements of cardiac function metrics indicate that function measurements from Sonic DL Cine-reconstructed images align well with those from fully sampled reference images. In conclusion, this study demonstrates that Sonic DL Cine is able to reconstruct highly under-sampled (up to 12-fold acceleration) cine datasets while preserving SNR, contrast, spatiotemporal sharpness, and quantification accuracy for cardiac function measurements. It also provides a feasible approach for thoroughly evaluating the deep learning-based method.

## 1. Introduction

Cardiac cine imaging is one of the most important components in routine clinical cardiac MR (CMR) exams [1,2]. Cine imaging has been widely adopted in clinical practice for assessing cardiac function such as chamber size, ejection fraction (EF), and wall motion abnormality [3,4]. The most commonly used cine sequence is two-dimensional (2D) balanced steady-state free precession (bSSFP) cine, which offers excellent signal-to-noise ratio (SNR) efficiency and blood–myocardium contrast [5,6], and allows clear, detailed visualization and measurement of cardiac structure and function. However, one main challenge of 2D bSSFP cine imaging is its relatively long scan time, even with parallel imaging techniques such as array coil spatial sensitivity encoding (ASSET), which requires patients to hold their breath during the scan to avoid respiratory or body motion artifacts. A typical CMR protocol [1] usually includes multiple 2D cine acquisitions on multiple cardiac views and multiple slices. Especially for short-axis acquisition, a stack of short-axis slices that cover the whole heart would be acquired for cardiac function measurement. This makes the whole cardiac MR exam time-consuming and challenging for patients with impaired breath-holding capacity or irregular heart rhythms. Additionally, inconsistent breath-holdings may lead to misalignment of cardiac anatomy on different slices and, thus, affect the following slice prescription and clinical interpretation [7]. Therefore, new techniques that enable faster cine acquisition are gaining more and more interest.

Simultaneous multi-slice (SMS) acquisition [8] was developed to accelerate 2D acquisition by using multiband radiofrequency pulses to excite more than one slice simultaneously and applying parallel imaging technique to reconstruct the superimposed slices. Although multiple attempts have been successfully made to accelerate cine acquisition with the SMS technique [9,10,11], it still suffers from reduced SNR and increased SAR burden. Another approach that has been explored to accelerate cine is the compressed sensing (CS) technique [12]. Previous studies have demonstrated the feasibility of the CS approach for accelerating cine acquisition in both healthy volunteers and various patient populations [13,14,15,16], but the reconstruction is computationally demanding due to the nonlinear and iterative reconstruction process [14] and prone to over-regularization and image blurring especially with high acceleration [17].

Recently, deep learning-based (DL-based) approaches have been explored to utilize prior knowledge learned from large databases for regularized image reconstruction of cine imaging [17,18,19,20,21,22]. These approaches typically involve multiple iterations of both conventional parallel imaging or CS algorithm and neural networks, with supervised training conducted in an end-to-end manner. Various neural network architectures, such as recurrent neural network (RNN) [19,23] and cascaded networks [18], have been explored, achieving up to nine-time acceleration for 2D cardiac cine imaging. Even higher acceleration can be achieved by combining aggressive under-sampling in kt space with a DL-based algorithm [17,20]. However, highly accelerated cine techniques often lead to degradation in image quality (e.g., blurring and aliasing artifact) [21,24]. Thorough evaluation of DL-based acceleration techniques for cine imaging is critical for potential clinical translations. Previous work [21] evaluated a DL-based reconstruction for bSSFP cine imaging through retrospective experiments with fully sampled short-axis cine datasets, investigating changes in image quality and quantification accuracy for measuring cardiac functions across acceleration factors up to eight. However, no evaluations were conducted on cine data from other cardiac planes (e.g., two-chamber, three-chamber, or four-chamber planes), which are crucial components in routine CMR exams. Additionally, the impact of DL-based reconstruction on the temporal sharpness of the cine images remains unclear when different acceleration factors are applied. Thus, systematic evaluations are still necessary to understand how acceleration factors and DL-based reconstruction impact the image quality and quantification accuracy for cardiac function measurements when accelerating the cine imaging.

In this study, we systematically evaluated Sonic DL Cine, a commercially available product application from GE HealthCare (Waukesha, WI, USA). This DL-based reconstruction technique is combined with variable-density (VD) under-sampling in kt space for accelerating cardiac cine imaging. Our hypothesis is that Sonic DL Cine can reconstruct under-sampled cine datasets with comparable image quality and quantification accuracy of cardiac function to fully sampled reference images across various acceleration factors. To test this hypothesis, we thoroughly investigated the effect of the acceleration factor on image quality both qualitatively and quantitatively with an MR-dedicated extended cardiac torso anatomical phantom (digital phantom) and fully sampled cine datasets from healthy volunteers on different cardiac planes. We also evaluated the impact of Sonic DL Cine on biventricular cardiac function measurements across different acceleration factors. Additionally, this study aims to provide a feasible retrospective approach for future evaluations of DL-based reconstruction techniques.

## 2. Methods

### 2.1. Deep Learning Model and Reconstruction Pipeline

**Figure 1:**
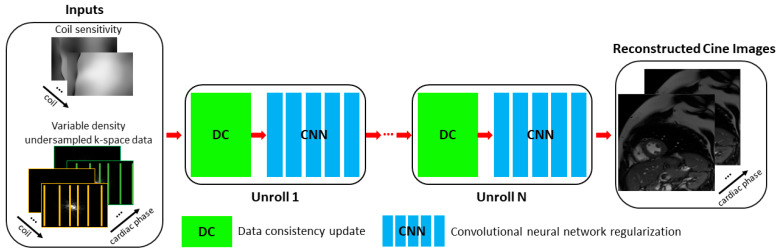
Reconstruction pipeline for Sonic DL Cine. The reconstruction pipeline for Sonic DL Cine takes a coil sensitivity map and multi-channel multi-phase under-sampled k-space cine data as inputs. The inputs go through multiple iterations of a data consistency (DC) step and a convolutional neural network (CNN) as regularization, until the final multi-phase cine images are reconstructed.

A specific DL-based reconstruction pipeline was developed for Sonic DL Cine to reconstruct the under-sampled 2D cine data. As illustrated in Figure 1, the pipeline follows a structure similar to previously reported design [17]. Multi-coil under-sampled 2D cine data and coil sensitivity maps are provided as inputs, which are used to compute initial zero-filled images across different cardiac phases and coil channels. These initialized images then undergo in total 12 iterations of reconstruction until the final multi-phase aliasing-free cine images are generated. For each iteration block, a physics-based data consistency (DC) step and image-based regularization using a convolutional neural network (CNN) are included. During the DC step, coil sensitivity maps are used to project the data back and forth between image and k-space domains, ensuring that the final output remains consistent with the acquired k-space data. The image-based regularization employs a CNN with 3D kernels to leverage all temporal and spatial dimensions information to remove under-sampling artifacts and noise [25].

For model training, fully sampled 2D cine datasets were retrospectively under-sampled using a kt-VD under-sampling scheme, as previously reported [26]. These under-sampled datasets were then input into the pipeline, which was trained in a supervised manner to reconstruct cine images free of aliasing artifacts. In total, 156 fully sampled bSSFP cine datasets from both 1.5 T and 3.0 T MR scanners (GE HealthCare, Waukesha, WI, USA) collected on multiple cardiac planes were included. Spatial cropping, flipping, and varied sampling patterns were applied to augment the training data. Mean absolute error (MAE) against the fully sampled data was used as the loss function during training. The Adam optimizer (beta1 = 0.9, beta2 = 0.999) with 1 × 10^−3^ as the learning rate was used in training, and the model training involves a total of 6.6 million trainable variables.

### 2.2. Evaluation with Digital Phantom

**Figure 2:**
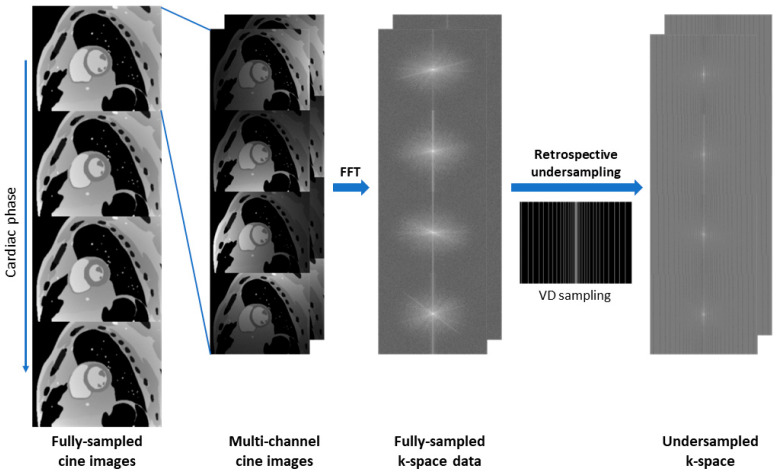
Pipeline of generating multi-channel under-sampled cine datasets from the digital phantom. Single-slice cardiac images with different cardiac phases are extracted from the digital phantom. The cine images then go through multi-channel simulation, fast Fourier transform (FFT), and retrospective under-sampling in kt space via a variable-density (VD) sampling scheme to generate multi-channel under-sampled k-space data.

An MR-dedicated extended cardiac torso (MRXCAT) anatomical phantom [27] was used to evaluate the performance of Sonic DL Cine. As shown in Figure 2, an 8-channel single-slice multi-phase cardiac cine complex dataset from the short-axis plane was generated from the MRXCAT phantom, which was then under-sampled in kt space with 4-fold, 8-fold, and 12-fold accelerations. The under-sampled k-space data were reconstructed using Sonic DL Cine and compared to corresponding fully sampled reference images. Peak SNR (PSNR), structural similarity index measure (SSIM), root mean square error (RMSE), and MAE were calculated for Sonic DL Cine-reconstructed images from different cardiac phases with the fully sampled data as reference. Additionally, to evaluate image spatiotemporal sharpness, a reference line was drawn across the whole heart on the fully sampled reference image at the end-systole phase, and signal profiles along the reference line across different cardiac phases were evaluated and compared to those from fully sampled images.

### 2.3. Evaluation with Healthy Volunteers

To further evaluate the performance of Sonic DL Cine, fully sampled cine datasets were acquired using a routine 2D bSSFP cine sequence on both 1.5 T and 3.0 T scanners (GE HealthCare, Waukesha, WI, USA) from healthy volunteers. All participants gave their written informed consent before the CMR scan. Retrospective cardiac gating was used to ensure that the full RR interval was acquired. A total of 40 datasets from healthy volunteers (*n* = 18) were included for the retrospective evaluation: 6 datasets from the short-axis plane, 11 datasets from the 2-chamber plane, 12 datasets from the 3-chamber plane, and 11 datasets from the 4-chamber plane. Detailed acquisition parameters are listed in Table 1. Retrospective under-sampled k-space data were generated using both the ASSET sampling pattern (acceleration factor = 2), which only retains alternate k-space lines, and the VD sampling pattern (acceleration factors = 4, 8, and 12), with a similar pipeline, as shown in Figure 2. The conventional parallel imaging technique ASSET served as a reference for assessing Sonic DL Cine. K-space data under-sampled with the kt-VD under-sampling scheme were reconstructed with Sonic DL Cine, and compared to ASSET with a uniform under-sampling scheme and fully sampled cine images. All datasets were reconstructed to contain 30 output cardiac phases using temporal interpolation to allow phase-by-phase comparison and downstream cardiac function measurement.

Once the images were reconstructed, image quality metrics including PSNR and SSIM were calculated for each cardiac phase for both ASSET and Sonic DL Cine images with the fully sampled images as reference. These metrics were then averaged over different cardiac phases for each subject for quantitative comparison. Representative images were compared for different cardiac views among ASSET, Sonic DL Cine, and fully sampled cine images. Additionally, to evaluate how Sonic DL Cine would affect image spatiotemporal sharpness, a reference line was drawn across the heart from a representative subject at the end-systole phase and signal profiles in both spatial and temporal domains were compared between the Sonic DL Cine images and fully sampled references.

### 2.4. Quantification of Cardiac Functions

In addition to image quality evaluations, tests were conducted to determine whether Sonic DL Cine impacts the accuracy of measuring cardiac function from short-axis stack cine images when different acceleration factors are applied. Measurements on fully sampled images served as reference. Biventricular volumetric indices, including left ventricle end-systole volume (LVESV), left ventricle end-diastole volume (LVEDV), left ventricle ejection fraction (LVEF), right ventricle end-systole volume (RVESV), right ventricle end-diastole volume (RVEDV), and right ventricle ejection fraction (RVEF), were measured from the fully sampled and Sonic DL Cine-reconstructed cine images on short-axis acquired from healthy volunteers (*n* = 6). The commercial software cvi42 (v5.1.4, Circle Cardiovascular Imaging, Calgary, AB, Canada), was used for automated post-processing and biventricular function measurement.

### 2.5. Statistical Analysis

Matlab (R2023b, The MathWorks Inc., Natick, MA, USA) was used for all statistical tests. Continuous variables were presented as means with standard deviations or as medians with interquartile ranges. Shapiro–Wilk test was used to verify normal distribution. Paired *t*-tests were performed to compare biventricular function measurements on Sonic DL Cine-reconstructed short-axis cine images against fully sampled references. Bland–Altman (BA) plots were used to evaluate the agreement of LV and RV volumetric indices measured from the two sets of cine images. A significance level of *p* < 0.05 was considered statistically significant.

## 3. Results

**Figure 3:**
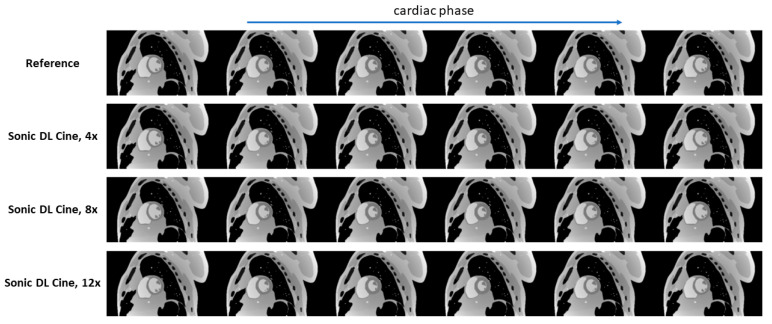
Reconstructed multi-phase cine images from the digital phantom. Multi-phase fully sampled cine images (reference) were retrospectively under-sampled by 4-fold, 8-fold, and 12-fold and reconstructed using Sonic DL Cine. PSNR, SSIM, RMSE, and MAE were calculated for images from each cardiac phase, and the mean values across different cardiac phases were reported. PSNR = peak signal-to-noise ratio; SSIM = structural similarity index measure; RMSE = root mean square error; MAE = mean absolute error.

**Figure 4:**
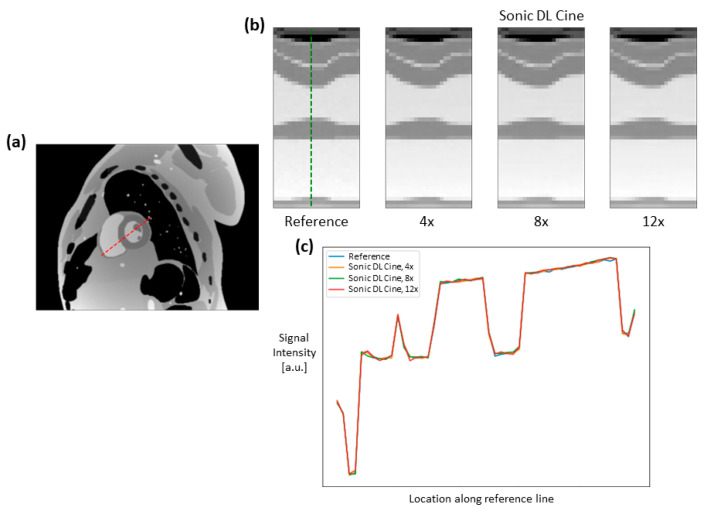
Spatiotemporal profiles of cine images from the digital phantom. A reference line (red dashed line in (**a**)) was drawn across the heart from the cine image at the end-systole phase. Signal profiles along the reference line through all cardiac phases from the fully sampled and Sonic DL Cine-reconstructed cine images (acceleration factors = 4, 8, and 12) are shown in (**b**). In particular, signal profiles at the end-systole phase (green dashed line in (**b**)) from different cine images are compared in (**c**).

Fully sampled and Sonic DL Cine-reconstructed cine images with different acceleration factors from the digital phantom are shown in Figure 3. Sonic DL Cine images with different acceleration factors generally exhibit comparable image quality to the fully sampled reference images across various cardiac phases. No difference in image contrast or sharpness is observed visually between Sonic DL Cine and fully sampled images. The corresponding metrics, PSNR, SSIM, RMSE, and MAE, averaged from different cardiac phases are reported in Table 2. The Sonic DL Cine images overall show high PSNR, and the SSIM, RMSE, and MAE values indicate high similarity between the Sonic DL Cine and fully sampled reference images, and the similarity drops slightly with increasing acceleration factor. Signal profiles along the reference line (red dashed line in Figure 4a) in both spatial and temporal domains are shown in Figure 4. Sonic DL Cine images with acceleration factors of 4, 8, and 12 show similar temporal sharpness as the fully sampled reference images (Figure 4b). Specially, in Figure 4c, the signal profile at the end-systole phase (green dashed line in Figure 4b) shows comparable image sharpness between Sonic DL Cine and fully sampled reference images even with an acceleration factor of up to 12. These results demonstrate that Sonic DL Cine can reconstruct high-quality cine images from the under-sampled k-space data with strong generalizability and robustness, given that the model has never seen non-MR data during training.

**Figure 5:**
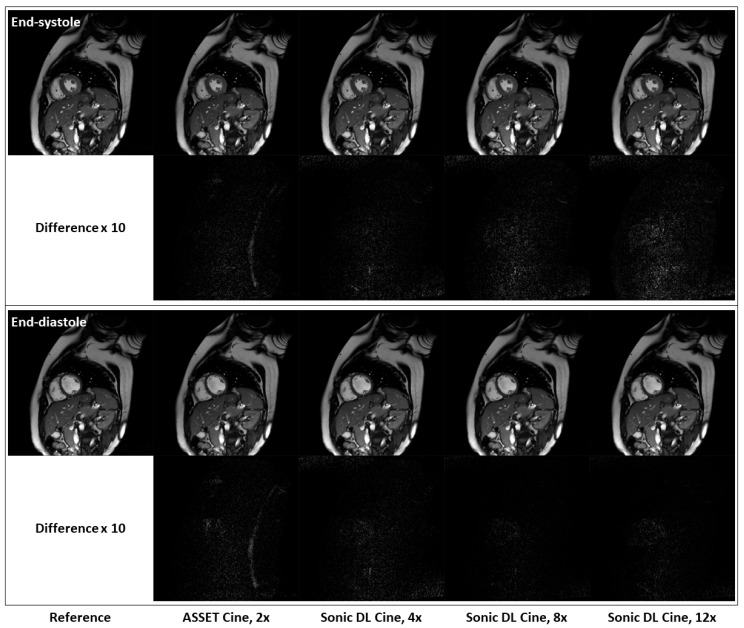
Representative fully sampled and retrospectively under-sampled cine images from the short-axis plane. Retrospectively under-sampled cine images with ASSET (acceleration factor = 2) and Sonic DL Cine (acceleration factors = 4, 8, and 12) are compared to the fully sampled reference at both end-systole and end-diastole phases. Difference maps, amplified by a factor of 10, between the under-sampled and fully sampled cine images are also calculated.

**Figure 6:**
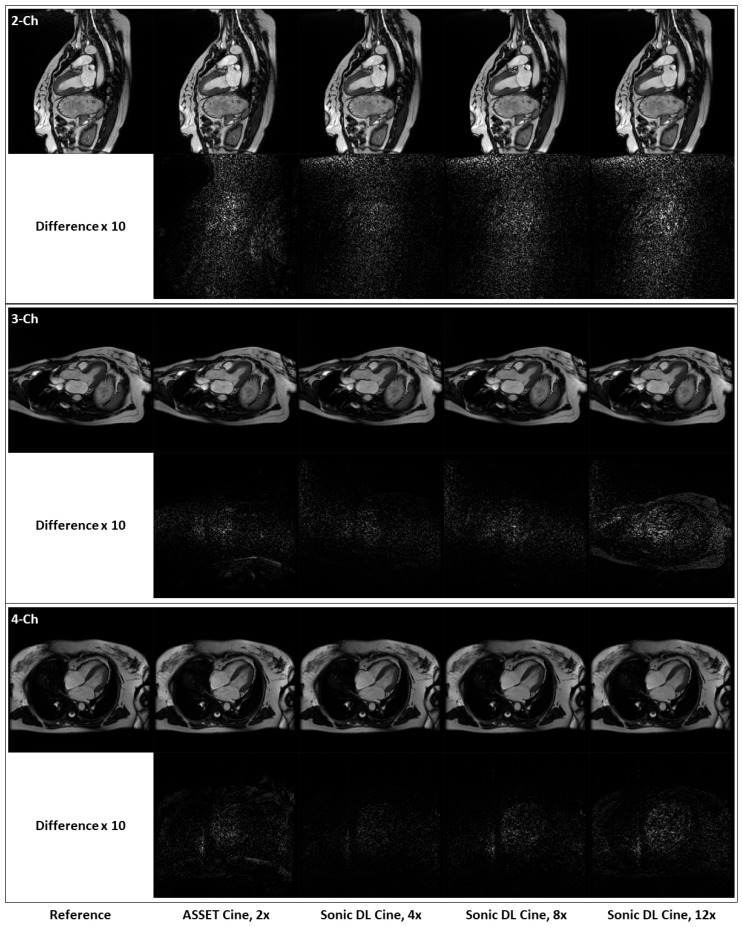
Representative fully sampled and retrospectively under-sampled cine images from the two-chamber, three-chamber, and four-chamber planes. Fully sampled and retrospectively under-sampled cine images with ASSET (acceleration factor = 2), and Sonic DL Cine (acceleration factors = 4, 8, and 12) on the two-chamber, three-chamber, and four-chamber planes are shown from top to bottom. Difference maps, amplified by a factor of 10, between the under-sampled and fully sampled cine images are also calculated.

Figure 5 shows fully sampled and retrospectively under-sampled cine images from a healthy volunteer in the short-axis plane. At both end-systole and end-diastole phases, Sonic DL Cine-reconstructed images exhibit comparable SNR, contrast, and sharpness to both ASSET (acceleration factor = 2) and fully sampled cine images. Difference maps between the under-sampled and fully sampled images also confirm that Sonic DL Cine can reconstruct high-quality cine images from highly under-sampled k-space data with minor discrepancies from the fully sampled reference images. The difference between the under-sampled and fully sampled images is more visible when high acceleration (e.g., 12-fold acceleration) is applied. Additionally, retrospectively under-sampled and fully sampled cine images acquired on two-chamber, three-chamber, and four-chamber planes from representative subjects are shown in Figure 6. From all these different planes, Sonic DL Cine images closely match ASSET (acceleration factor = 2) and fully sampled cine images in terms of SNR, sharpness, and contrast, which is also demonstrated by difference maps between under-sampled and fully sampled images. Increased acceleration factors make the difference between Sonic DL Cine and fully sampled reference images more visible.

**Figure 7:**
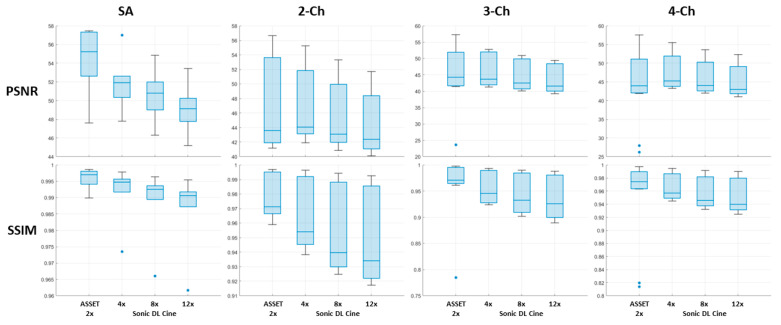
Quantitative evaluation of image quality for retrospectively under-sampled cine images from the short-axis, two-chamber, three-chamber, and four-chamber planes. PSNR and SSIM (with fully sampled cine images as reference) are calculated for retrospectively under-sampled cine images acquired on the short-axis, two-chamber, three-chamber, and four-chamber planes from all subjects. The central box represents the interquartile range (IQR), which contains the middle 50% of the data. It is bounded by the first and third quartiles. The line inside the box indicates the median value, and the lines extending from the box show the range of the data outside the IQR, which extend to the smallest and largest values within the range of 1.5 × IQR from the first and third quartiles. The stars are outliers outside the 1.5 × IQR range.

**Figure 8:**
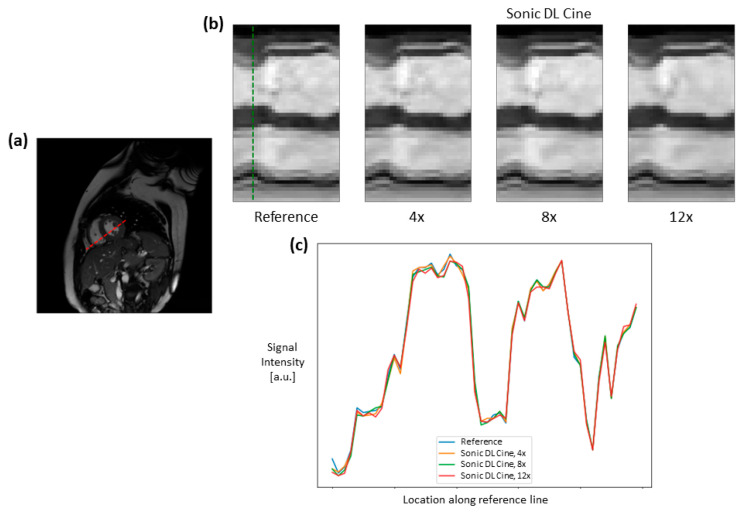
Spatiotemporal profiles of cine images from healthy volunteers on the short-axis plane; (**a**) shows the reference line (red dashed line) drawn across the heart from the cine image at the end-systole phase. Signal profiles along the reference line across all cardiac phases for the fully sampled and Sonic DL Cine-reconstructed cine images (acceleration factors = 4, 8, and 12) are shown in (**b**). In particular, signal profiles at the end-systole phase (green dashed line in (**b**)) from Sonic DL Cine and reference images are compared in (**c**).

To quantitatively evaluate the image quality of the under-sampled cine images compared to fully sampled images, PSNRs and SSIMs were calculated from different cardiac views for all healthy subjects, as summarized in Figure 7. In general, Sonic DL Cine-reconstructed images with 4-fold, 8-fold, and 12-fold accelerations show comparable PSNR to ASSET cine images with 2-fold acceleration and a median above 40 dBs, even though the PSNR drops slightly when Sonic DL Cine acceleration increases. SSIM values demonstrate high similarity between Sonic DL Cine and fully sampled images. Although the SSIMs are slightly lower than those of ASSET cine images and decrease as the Sonic DL Cine acceleration factor increases, the similarity remains generally high for Sonic DL Cine images, even with 12-fold acceleration. Additionally, signal profiles along the reference line (red dashed line in Figure 8a) in both spatial and temporal domains are shown in Figure 8. Sonic DL Cine images with any acceleration factor show similar temporal sharpness as the fully sampled reference images (Figure 8b). Specifically, as shown in Figure 8c, the signal profiles at the end-systole phase (green dashed line in Figure 8b) confirm comparable spatial sharpness between Sonic DL Cine images with 4-fold and 8-fold accelerations and the fully sampled images, and subtle blurring is observed from Sonic DL Cine images with 12-fold acceleration.

**Figure 9:**
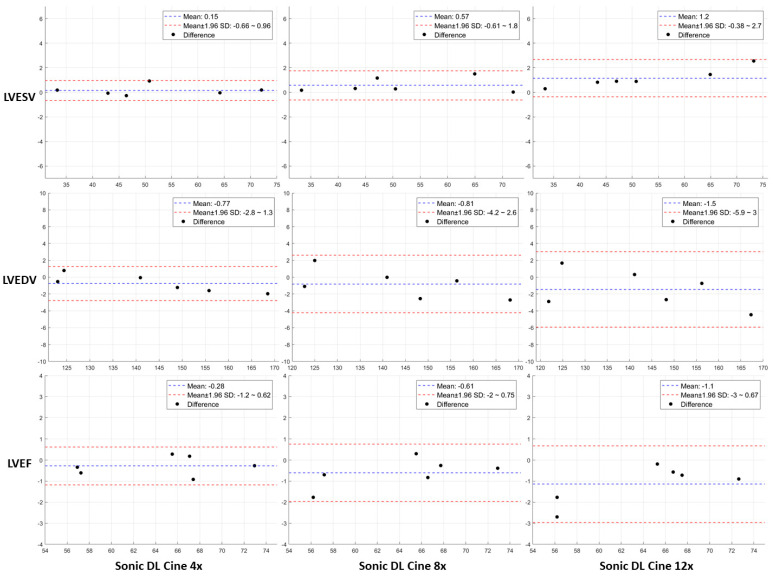
Comparison of left ventricular cardiac function measured from Sonic DL Cine-reconstructed and fully sampled cine images on the short-axis plane. LVESV, LVEDV, and LVEF were measured from fully sampled and Sonic DL Cine-reconstructed cine images (acceleration factors = 4, 8, and 12), and Bland–Altman plots are drawn to assess the agreement between measurements from the two sets of data. Each point represents the bias between measurements from the Sonic DL Cine and fully sampled cine images for each subject. The dashed blue line shows the mean bias between measurements, and the dashed red lines represent the upper and lower 95% limits of agreement. LVESV = left ventricle end-diastolic volume; LVEDV = left ventricle end-systolic volume; LVEF = left ventricle ejection fraction.

**Figure 10:**
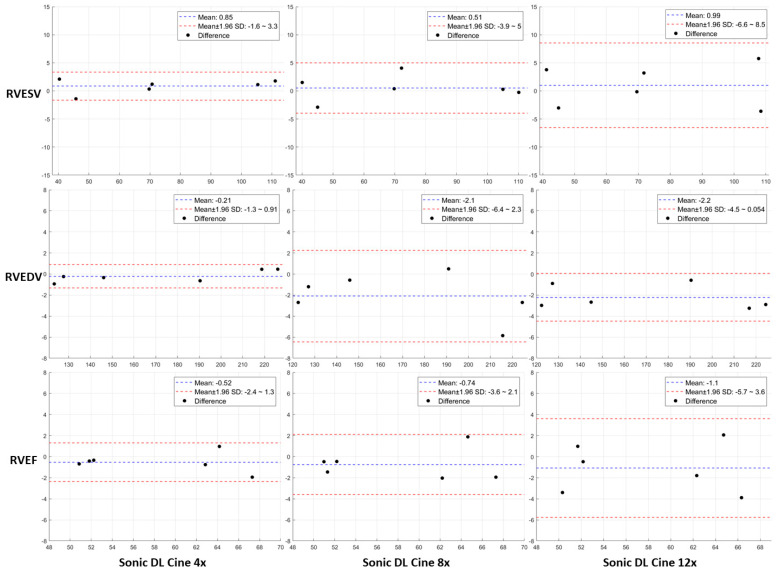
Comparison of right ventricular cardiac functions measured from Sonic DL Cine-reconstructed and fully sampled cine images on the short-axis plane. RVESV, RVEDV, and RVEF were measured from fully sampled and Sonic DL Cine-reconstructed cine images (acceleration factors = 4, 8, and 12), and Bland–Altman plots are drawn to assess the agreement between measurements from the two sets of data. Each point represents the bias between measurements from the Sonic DL Cine and fully sampled cine images for each subject. The dashed blue line shows the mean bias between measurements, and the dashed red lines represent the upper and lower 95% limits of agreement. RVESV = right ventricle end-diastolic volume; RVEDV = right ventricle end-systolic volume; RVEF = right ventricle ejection fraction.

To further evaluate the performance of Sonic DL Cine, biventricular cardiac function was measured and compared between the Sonic DL Cine-reconstructed and fully sampled short-axis cine images. The results are summarized in Table 3. When 4-fold and 8-fold accelerations were applied, no significant differences in the volumetric indices were observed between measurements from Sonic DL Cine and fully sampled cine images for both left and right ventricles (*p* > 0.05). However, with a 12-fold acceleration, statistically significant differences were found in LVESV (*p* = 0.02), LVEF (*p* = 0.03), and RVEDV (*p* = 0.01) measurements between Sonic DL Cine and fully sampled images, likely due to the image blurring seen in Figure 8c. No significant differences were found in the measurement of LVEDV, RVESV, and RVEF from the two sets of cine images (*p* > 0.05) when 12-fold acceleration was applied. The agreements in measuring biventricular cardiac function from Sonic DL Cine-reconstructed and fully sampled cine images are shown in BA plots in Figure 9 and Figure 10, respectively. For both LVESV and LVEDV, less than a 1.5 mL difference is observed between the measurements from the two sets of cine images with varying acceleration factors. For LVEF, the differences between measurements from Sonic DL Cine and fully sampled images are less than 1.1%. For the right ventricle, less than 2.2 mL differences are observed in RVESV and RVEDV measured from fully sampled and Sonic DL Cine images with different acceleration factors. For RVEF, differences between measurements from the Sonic DL Cine and fully sampled images are less than 1.1%. Overall, for both left and right ventricle function, increasing the Sonic DL Cine acceleration factor slightly raises the bias and variability between measurements from Sonic DL Cine and fully sampled images.

## 4. Discussion

This study systematically evaluated the performance of Sonic DL Cine in a retrospective manner, across various acceleration factors and cardiac views. This approach eliminates the influence of system imperfections and scan-to-scan variations that can occur in prospective experiments. Additionally, by using fully sampled data as a reference, objective comparisons become feasible, allowing for the use of quantitative metrics such as SSIM to efficiently assess Sonic DL Cine. MRXCAT digital phantom experiment helps eliminate physiological-related variances, making it a powerful and flexible test tool for assessing performance and robustness of Sonic DL Cine.

The results from both digital phantom and in vivo experiments demonstrate that Sonic DL Cine effectively reconstructs highly accelerated cine images with strong generalizability and robustness. Qualitative and quantitative comparisons on the digital phantom and healthy volunteer data confirm comparable SNR, contrast, and spatiotemporal sharpness to the fully sampled reference images, despite slight degradations in image quality (particularly image sharpness) when a high acceleration factor (e.g., 12-fold) was applied. Furthermore, Sonic DL Cine had negligible impact on measuring biventricular cardiac function when low-to-moderate accelerations (e.g., 4-fold to 8-fold) were used, compared to the fully sampled reference images. However, when 12-fold acceleration was applied, statistically significant differences were observed in the measurements of LVESV, LVEF, and RVEDV (*p* < 0.05) between Sonic DL Cine-reconstructed and fully sampled cine images. These differences are likely due to image blurring seen in short-axis cine images with 12-fold acceleration, which potentially affects accuracy of the automatic segmentation with cvi42.

### 4.1. Retrospective vs. Prospective Evaluation

Despite the advantages of retrospective experiments, there are still limitations when compared to prospective evaluations. One key limitation is that retrospective studies cannot replicate the complex clinical setting, such as the variability introduced by the MR system, the operator, or the patient. These factors could potentially impact the performance of Sonic DL Cine and require further investigation. Specifically, considering the gender, age, and various pathological conditions of patients, it would be valuable to conduct prospective evaluations of Sonic DL Cine across different patient populations. Additionally, a fixed protocol was used for the data acquisition in the retrospective experiments. Although this protocol closely matches actual clinical protocols, it cannot capture all the acquisition variances that may arise with different patients. In clinical practice, acquisition parameters are often tailored to each patient to ensure optimal image quality, and this can only be validated through prospective experiments. Moreover, retrospective studies cannot evaluate the technique’s performance on the impact of the contrast agent or contrast dynamics on the technique.

Several prospective clinical studies have been conducted to date, demonstrating the feasibility and robustness of Sonic DL Cine across different populations. For example, Eyre K, et al. have demonstrated that Sonic DL Cine with 8-fold acceleration achieved a 37% reduced acquisition time without significantly affecting image quality or volumetry with both healthy volunteers and patients [28]. Other studies have reached similar conclusions in pediatric populations [20,29] and adult patients [30,31,32,33,34]. All of these prospective clinical studies offer strong evidence that Sonic DL Cine, with moderate acceleration, significantly reduces scan time while delivering comparable or even superior image quality. Additionally, it maintains accuracy in cardiac function and volume measurements when compared to conventional bSSFP cine with parallel imaging.

### 4.2. Potential Clinical Utilization of Sonic DL Cine

The results of this study demonstrate that Sonic DL Cine maintains consistent performance, with only a slight decrease in image quality as the acceleration factor increases, suggesting that it can be used across a broad range of acceleration factors. Low-to-moderate accelerations are suitable when prioritizing high image quality while also reducing scan time. For cases where significantly reduced scan time is necessary, such as for patients with ischemic heart disease, high acceleration (e.g., 12-fold acceleration) can be used. Specifically, with 12-fold acceleration, cine acquisition for each slice can be completed within a single RR interval, enabling rapid free-breathing and arrhythmia-robust imaging. A user-prescribed arrhythmia acceptance window can be added to ensure that only desired RR intervals are acquired. Previous studies have demonstrated the use of Sonic DL Cine with 12-fold acceleration for real-time respiratory-gated [30] and free-breathing cine acquisition [31], concluding that Sonic DL Cine provides comparable image quality and reliable cardiac function measurements when compared to the standard breath-hold bSSFP cine sequence.

Additionally, Sonic DL Cine can be integrated into post-contrast cine acquisition to further utilize the non-scanning time prior to late gadolinium enhancement imaging. A previous study [35] has shown that the diagnostic performance of Sonic DL Cine is equivalent for both pre- and post-contrast injections in assessing image quality as well as biventricular volumes and functions.

### 4.3. Limitations and Future Work

The current study has several limitations. Firstly, the digital phantom only simulates a short-axis view with certain contrast and heart rate. Secondly, the in vivo evaluation was conducted with a relatively small cohort, primarily due to the challenges in acquiring high-quality fully sampled in vivo data. Thirdly, all in vivo data were obtained from healthy adult volunteers, and there is a lack of fully sampled data from patients (e.g., those with congenital heart disease) or pediatric subjects. In the future, enhancing digital phantom simulation to include more variations such as contrast change, cardiac view, or arrhythmic heart beat could help evaluate accelerated MR techniques more comprehensively. Furthermore, for the in vivo evaluation, retrospective studies using data collected with clinical standards and mild accelerations will be explored to make data collection for evaluation more feasible. Furthermore, retrospective studies involving larger patient and pediatric cohorts will be conducted to further assess the performance of Sonic DL Cine.

## 5. Conclusions

In conclusion, we conducted a comprehensive retrospective study to evaluate a DL-based cine sequence, Sonic DL Cine. Evaluations on both MRXCAT digital phantom and healthy volunteer data demonstrate that Sonic DL Cine is able to reconstruct highly under-sampled cine datasets (up to 12-fold acceleration) with comparable SNR, contrast and spatiotemporal sharpness to the fully sampled reference images. The biventricular function measurements demonstrate that Sonic DL Cine has negligible impact on measuring cardiac function, especially when low-to-moderate accelerations are applied. This study also offers a feasible approach for future systematic evaluations of DL-based reconstruction techniques.

## Figures and Tables

**Table 1:** Acquisition parameters for fully sampled bSSFP cine imaging in healthy volunteers.

MR Parameter	Value
XRES	192–200
YRES	192–224
TE/TR [ms]	Min Full/3.0–4.5
FOV [cm]	36–40
BW [Hz]	90–125
Slice thickness [mm]	8

**Table 2:** Image quality evaluation of retrospectively under-sampled cine images with Sonic DL Cine reconstruction compared to the fully sampled reference images from the digital phantom.

Acceleration Factor	PSNR	SSIM	RMSE	MAE
4	46.65	0.95	0.0047	0.0037
8	45.48	0.94	0.0053	0.0042
12	44.76	0.94	0.0058	0.0045

**Table 3:** Summary of biventricular cardiac function measured from Sonic DL Cine-reconstructed and fully sampled cine images on the short-axis. (*p*: *p*-value from the paired *t*-test comparing measurements between Sonic DL Cine and fully sampled cine images).

	Fully Sampled	Sonic DL Cine, 4×	Sonic DL Cine, 8×	Sonic DL Cine, 12×
LVESV [mL]	51.53 ± 14.24	51.68 ± 14.26 (*p* = 0.41)	52.11 ± 14.36 (*p* = 0.07)	52.68 ± 14.98 (*p* = 0.02)
LVEDV [mL]	143.98 ± 18.32	143.21 ± 17.43 (*p* = 0.13)	143.17 ± 17.21 (*p* = 0.31)	142.52 ± 17.20 (*p* = 0.18)
LVEF [%]	64.65 ± 6.25	64.37 ± 6.34 (*p* = 0.19)	64.04 ± 6.67 (*p* = 0.08)	63.51 ± 6.96 (*p* = 0.03)
RVESV [mL]	73.42 ± 29.18	74.27 ± 29.62 (*p* = 0.16)	73.93 ± 29.34 (*p* = 0.61)	74.41 ± 29.37 (*p* = 0.56)
RVEDV [mL]	172.16 ± 45.50	171.95 ± 45.93 (*p* = 0.41)	170.06 ± 44.63 (*p* = 0.07)	169.94 ± 45.23 (*p* = 0.01)
RVEF [%]	58.47 ± 7.44	57.95 ± 7.29 (*p* = 0.23)	57.73 ± 7.50 (*p* = 0.27)	57.39 ± 7.33 (*p* = 0.32)

## Data Availability

The original contributions presented in this study are included in the article. Further inquiries can be directed to the corresponding author.
